# The use of music in nursing care for psychological distress reduction in cancer patients: A scoping review protocol

**DOI:** 10.1371/journal.pone.0335895

**Published:** 2025-11-24

**Authors:** Karena Cristina da Silva Leal, Maria Améllia Lopes Cabral, Myrza Torres Ferreira, Danielle de Oliveira Rocha, Lara Dantas de Rubim Costa, Geovana Caroliny de Morais Silva, Louise Constância de Melo Alves Silva, Daniele Vieira Dantas, Rodrigo Assis Neves Dantas

**Affiliations:** 1 Postgraduate Program in Nursing, Federal University of Rio Grande do Norte (UFRN), Natal, Rio Grande do Norte, Brazil; 2 Nursing Graduate Program, Federal University of Rio Grande do Norte (UFRN), Natal, Rio Grande do Norte, Brazil; Federal University of Paraiba, BRAZIL

## Abstract

Psychological distress in cancer patients is a frequent and complex condition that significantly compromises quality of life, from diagnosis to palliative care. In this context, the use of music emerges as a promising, safe, and low-cost complementary practice capable of alleviating symptoms such as emotional distress. However, there are still gaps in the literature regarding the types, contexts, and specific effects of musical interventions in cancer patients. Thus, this protocol proposes a scoping review to map the available scientific evidence on the use of music as an intervention for the reduction of psychological distress in cancer patients. The methodology will adhere to the guidelines of the Joanna Briggs Institute and the Preferred Reporting Items for Systematic Reviews and Meta-Analyses Extension for Scoping Reviews (PRISMA-ScR) checklist. The search will be conducted across twenty data sources, including PubMed Central, Latin American and Caribbean Literature in Health Sciences, Cumulative Index to Nursing and Allied Health Literature, Web of Science, Scientific Electronic Library Online, Cochrane Library, Elsevier’s Scopus, ScienceDirect, Wiley Online Library, and the Virtual Health Library. For gray literature, Catalog of Theses and Dissertations of the Coordination for the Improvement of Higher Education Personnel, the Networked Digital Library of Theses and Dissertations,Google Scholar, European Thesis Portal; Electronic Thesis Online Service; Open Access Scientific Repository of Portugal; National Theses and Dissertations; Theses Canada; Treasury of the National Library of Australia; Online Academic Archive will be searched. Study screening and selection will be performed independently by two researchers according to the inclusion and exclusion criteria. Relevant data will be extracted into a structured spreadsheet, including study characteristics, type and setting of the musical intervention, outcomes related to psychological distress, and the role of nursing. The findings are expected to inform clinical practice, particularly in the field of oncology nursing, as well as to identify gaps for future research.

## Introduction

Cancer ranks as the second leading cause of death, representing one of the greatest public health challenges, with statistics indicating a growing increase in incidence and mortality globally each year [1]. It is estimated that the lifetime risk of developing any type of neoplasm is 40% for men and 38% for women, and this global burden also significantly impacts healthcare systems [2].

Although more than one hundred types of malignant neoplasms have been identified, certain anatomical sites show higher prevalence depending on gender. Among men, prostate cancer is the most common, followed by cancers of the colon and rectum, lung, stomach, and oral cavity. Among women, breast cancer predominates, followed by cancers of the colon and rectum, cervix, lung, and thyroid, in decreasing order of incidence [3].

Cancer staging is determined by the tumor-node-metastasis (TNM) system, which assesses the extent of the disease by considering tumor size and characteristics, lymph node involvement, and the presence of metastases, allowing identification of the spread of malignant lesions. Based on this assessment, neoplasms are classified into stages ranging from zero, when confined to the site of origin, to four, when there is dissemination to adjacent tissues or distant organs [4].

Staging is, therefore, a fundamental step in the diagnostic process, as it guides and standardizes therapeutic approaches. The main treatment modalities include surgery, chemotherapy, and radiotherapy, which are usually employed in combination, although they may occasionally be used alone [5].

A cancer diagnosis can evoke intense suffering encompassing emotional, physical, and social dimensions [2]. In this context, it is characterized as a feared and impactful moment in an individual’s life, affecting not only the diagnosed person but also their involved family circle. Among the most recurrent feelings are the fear of death, disease recurrence, anxieties about treatment, changes in affective bonds, and the economic implications that often accompany the illness process [6].

Psychological distress is a broad construct encompassing negative emotions and feelings that can impair an individual’s overall functioning and interfere with daily activities. This condition may distort self-perception and the understanding of one’s environment, manifesting through sadness, anxiety, inattention, and other symptoms related to mental health. Among individuals with cancer, psychological distress tends to have multidimensional repercussions, affecting financial, family, sexual, spiritual, and social aspects of life.

Consequently, patients undergoing chemotherapy frequently experience multiple adverse effects that compromise their well-being [7]. Evidence indicates that individuals with cancer may present with more than ten concurrent symptoms related to the disease and its treatment, underscoring the complexity of oncological care and the need for complementary interventions to mitigate multifactorial suffering [8].

Recent research indicates that psychological distress can act as a catalyst and negatively influence cancer prognosis, being associated with higher mortality and lower survival rates. Thus, emotional distress has been recognized as the “sixth vital sign” in oncology, underscoring the importance of management and relief strategies throughout treatment [9].

Non-pharmacological interventions have gained prominence as complementary approaches to alleviate treatment-related discomfort and improve patient outcomes. Strategies such as cognitive-behavioral therapy, yoga, distraction techniques, virtual reality, music therapy, and progressive muscle relaxation have demonstrated benefits, with the latter two standing out for their feasibility, low cost, and positive acceptance in clinical practice [10].

The integration of psychosocial interventions, such as music therapy, into comprehensive oncological care effectively addresses not only physical symptoms but also promotes improvements in patients’ psychological and emotional states. Evidence highlights that adherence to alternative strategies contributes to overall well-being, fostering quality of life and coping abilities in the face of treatment and illness [11].

Musical interventions have been shown to reduce anxiety by modulating stress-related hormones and inducing physiological states of calm and relaxation. They influence the autonomic nervous system, helping stabilize respiratory rate, heart rate, blood pressure, and pulse [12,13].

In general, musical interventions involve listening to pre-selected music, guided or facilitated by healthcare professionals. Music therapy, in contrast, entails a structured therapeutic process conducted by a qualified music therapist, who uses individualized musical experiences to achieve specific clinical objectives [14].

Within this context, nursing care seeks to address the biopsychosocial and spiritual needs of individuals through preventive, promotive, and rehabilitative actions grounded in ethical, aesthetic, empirical, personal, and political ways of knowing. Thus, music emerges as a complementary strategy in nursing, offering therapeutic benefits while fostering sensitivity and humanization in care practice [15–17].

Nevertheless, despite existing studies on the use of music in oncological contexts, the scientific output remains fragmented regarding approaches focused on the psychological distress experienced by these patients. Therefore, this manuscript is justified by its contribution to the scientific community, showcasing studies that address the use of music for reducing psychological distress in cancer patients and expanding knowledge about this practice, identifying existing literature gaps, and contributing to scientific advancement in the field.

A search was conducted to identify reviews with similar themes, ensuring the exclusivity of the data. The Open Science Framework (OSF) platform was searched, and no publications with a scope similar to this review were found. Thus, this study aims to map how music has been used in nursing care to alleviate psychological distress among adult cancer patients in oncology rehabilitation settings.

## Methods

This scoping review protocol is conducted based on the Preferred Reporting Items for Systematic Reviews and Meta-Analyses Extension for Scoping Reviews (PRISMA-ScR) [18] and the guidelines formulated by the Joanna Briggs Institute (JBI) [19]. This study was pre-registered on the Open Science Framework (OSF) public platform prior to commencing the formal search (https://osf.io/vxazq/). Complementary searches were also conducted in the international repositories PROSPERO and the Cochrane Library, where no registered protocols with identical themes were identified, reinforcing the originality of the present study.

The work was developed following the methodological framework proposed by Arksey & O’Malley (2005) [20], which was subsequently expanded, adhering to the following stages: 1) defining the research question; 2) aligning the review’s inclusion and exclusion criteria; 3) approaching study selection; 4) selecting, extracting, and presenting evidence; 5) analyzing extracted data; 6) presenting results; and 7) synthesizing data according to the objective.

### Research question

The Population, Concept, and Context (PCC) mnemonic established by the JBI was used to define the research question. The Population was defined as: Adult cancer patients; Concept: Use of music in nursing care as a therapeutic intervention for the reduction of psychological distress; Context: Oncological rehabilitation institutions. Based on this framework, the following research question was formulated to guide the study: “How has music been used in nursing care to alleviate psychological distress among adult cancer patients in oncology rehabilitation settings?”

### Eligibility criteria

In this regard, the following inclusion criteria were employed: studies available in the utilized databases that addressed the theme, were accessible in full text, with no temporal or language restrictions. Conversely, the following were excluded: opinion articles, letters to the editor, editorials; studies involving pediatric or adolescent populations; studies of patients with cancers affecting auditory perception or cognitive function; studies not using music as the primary intervention; studies conducted outside oncology rehabilitation institutions or hospitals; and studies that did not address the research question.

### Search strategy

Initially, a limited preliminary search was performed in the PubMed and CINAHL databases to identify descriptors, keywords, and indexing terms frequently used in studies related to the scope review to be developed.

Following the initial analysis of studies in the databases, a consultation was conducted with the Health Sciences Descriptors (DeCS) and Medical Subject Headings (MeSH), with adaptations for language (Portuguese and English) to optimize results. The following indexed descriptors were used: “Music Therapy”, “Psychological Distress”, “Anxiety”, “Depression” “Subjective Stress”, “Nursing Care”, “Neoplasms”. The Boolean operators “AND” and “OR” were used to combine the descriptors in the search strategy.

The databases were selected based on their comprehensiveness and relevance to the field of healthcare and music therapy in oncology, including internationally indexed databases that concentrate clinical studies, systematic reviews, and literature on musical interventions.

To complement the search and minimize publication bias, gray literature sources, including dissertations, theses, and institutional reports, will be considered, alongside reverse search procedures (checking the references of included studies) to identify additional relevant evidence. No restrictions will be applied regarding language or publication date. Studies published in languages other than Portuguese or English will be translated or analyzed with the assistance of specialized translators, and all publication dates will be included to capture the full extent of available evidence for gray literature. This approach broadens the scope of the review, ensuring that all pertinent evidence, regardless of language or date, is incorporated into the scoping summary.

Twenty data sources will be used to construct this review, namely: PubMed Central (PMC), Latin American and Caribbean Health Sciences Literature (LILACS), Cumulative Index to Nursing and Allied Health Literature (CINAHL), Web of Science, Scientific Electronic Library Online (SciELO), Cochrane Library, Elsevier’s Scopus, ScienceDirect, Wiley Online Library, and Virtual Health Library (BVS). For gray literature, the Catalog of Theses and Dissertations of the Coordination for the Improvement of Higher Education Personnel (CAPES), Networked Digital Library of Theses and Dissertations (NDLTD), Google Scholar, European Thesis Portal (DART); Electronic Thesis Online Service (EThOS); Open Access Scientific Repository of Portugal (RCAAP); National Theses and Dissertations (ETD Portal); Theses Canada; Treasury of the National Library of Australia (TROVE); Online Academic Archive (DIVA). The search will be conducted through the CAPES Periodicals Portal, via remote access on the Federated Academic Community (CAFe) platform. As an example of the search strategy the following string was created for the PubMed PMC database, represented in Table 1, resulting in 1964 free full-text documents in October 2025.

**Table 1:** Search strategy in PubMed PMC. Natal, RN, Brasil, 2025.

Database	Search Strategy
Pubmed PMC	((((((Music Therapy) AND (Psychological Distress)) OR (Anxiety)) OR (Subjective Stress)) OR (depression)) AND (neoplasm)) AND (nursing care)

Source: Authors, 2025.

The literature search is scheduled to be conducted primarily in November 2025, including all articles published up to that date. The subsequent stages of the study are expected to be completed by the end of December or January 2025.

### Study selection

The screening of publications will be performed independently by two researchers. After reading the titles and abstracts, considering the inclusion and exclusion criteria, any discrepancies during the article selection process will be resolved by consulting a third researcher. It is important to note that after study selection, data extraction aims to include relevant data for obtaining the expected results of the scoping review. Subsequently, after full-text article selection, studies that answer the research question will be included in the final sample.

To optimize the screening process and ensure transparency, Rayyan software will be employed. This tool allows the import of records from multiple databases and gray literature sources, systematically identifying and removing duplicates. In addition, it enables two reviewers to independently screen titles and abstracts, automatically recording disagreements and flagging studies with incomplete or pending data, thereby ensuring traceability and comprehensive documentation of the selection process.

### Analysis of results

Study data will be collected using a structured form in an Excel spreadsheet with the following variables: principal author, country, year of publication, study type, database where the study was found, sex, type of cancer, disease stage, study objective, intervention setting, indicators and scales used, limitations, conclusions, and relevance to nursing practice.

The methodological quality of the included studies will be assessed using the Joanna Briggs Institute (JBI) critical appraisal instruments, applied according to each study design (e.g., randomized controlled trials, cross-sectional studies, cohort studies, qualitative studies, or mixed methods studies). This assessment will not be used to exclude studies but will enhance methodological rigor and transparency in interpreting the findings. Results will be presented in a summary table, including author and year of publication, study design, checklist used, quality rating (high, moderate, or low), and relevant observations regarding limitations or methodological aspects affecting the reliability of the results. This approach allows readers to quickly understand the robustness of the included studies without compromising the clarity of the scoping review report.

To address the expected heterogeneity of interventions and outcomes, extracted data will be organized thematically. Studies will be categorized according to: (1) type of musical intervention (music therapy, music listening, or other), (2) intervention setting (hospital, outpatient, home, or virtual), (3) main outcomes related to psychological distress (e.g., anxiety, depression, stress), and (4) the role of nursing in implementing the intervention. This thematic structure will enable a clear synthesis of diverse findings and facilitate the identification of patterns, gaps, and implications for nursing practice. Data will be summarized in tables and charts to provide a transparent and accessible overview aligned with the study objectives and research question.

Issues related to missing data in the included studies will be addressed transparently. All incomplete information will be recorded and described in the summary of results, ensuring that missing data are clearly documented and considered in interpreting the findings.

The scoping review will follow the PRISMA-ScR recommendations, ensuring transparency and standardization in the search, selection, and data extraction process, as illustrated in Figure 1 in the appendices [18]. Additionally, the PAGER framework will be adopted for presenting and synthesizing results, organizing evidence into Patterns, Advances, Gaps, and Evidence for Practice. This integration combines methodological rigor in conducting the review (PRISMA-ScR) with clarity and objectivity in communicating findings and identified gaps (PAGER), avoiding redundancies and facilitating interpretation [21].

Additionally, the PRISMA flowchart [15] will be completed to describe the selection and inclusion of results, (see S1 Fig in the Supporting Information), and the PAGER methodology will be used to enhance reporting quality, thereby providing greater methodological rigor to the scoping review. The PAGER framework is a tool for scoping reviews, an approach to analyze the review through the following elements: Patterns, Advances, Gaps, Evidence for practice, and Research recommendations [21].

## Discussion

The susceptibility to experiencing psychological distress can accompany cancer patients at various stages, from diagnosis to palliation, irrespective of age. Statistics indicate that the likelihood of a cancer patient developing psychoemotional symptoms is double that of a healthy individual, remaining an inherent reality for the patient even after treatment cessation and disease remission [22]. Mental suffering from treatment, combined with the emotional burden of a cancer diagnosis, compromises patients’ quality of life and increases suicide risk, highlighting the need for integrative approaches to address the psychological dimension of coping with cancer [10].

Complementary non-pharmacological strategies have been widely investigated for their potential to promote more humanized and holistic care, contributing significantly to the alleviation of stress, anxiety, and other emotional symptoms in patients. Thus, nursing, grounded in a holistic perspective, plays a leading role in the use of effective non-pharmacological integrative and complementary practices for reducing pain, anxiety, nausea, and other symptoms [23].

Music therapy, as a rising non-pharmacological strategy, has been applied in the context of oncological patient care. Pre-existing systematic reviews indicate that the use of this approach promotes positive effects in reducing anxiety and depression. Research conducted in a palliative care setting revealed that patients, when immersed in the experience of musical intervention, direct their attention to pleasant experiences. This participation contributes to the alleviation of suffering and assists the patient in overcoming the frequent difficulty of verbally expressing emotions and feelings [24,25].

In a study conducted by Lagattolla F. *et al*., it was demonstrated that music can induce relaxation in breast cancer patients, contributing to the reduction of anger, heart and respiratory rates, as well as attenuating pain, decreasing the need for anesthetics and analgesics, and shortening recovery time—factors that significantly contribute to the reduction of anxiety and depression [26].

In this sense, it is observed that interventions aimed at spiritual well-being are not yet comprehensively addressed in clinical practice, which can negatively impact treatment adherence, quality of life, and even functional recovery of patients. Therefore, the production of empirical evidence supporting practices capable of promoting physical health, psychosocial adaptation, and spiritual balance constitutes a fundamental priority in the context of healthcare provision [25].

In summary, this scoping review will map current evidence on musical interventions in the psychosocial care of cancer patients, providing nursing professionals with up-to-date knowledge to support effective and compassionate practice. It may also inform the development of structured intervention protocols and stimulate further research in this area.

## Conclusions

This scoping review protocol aims to map the available scientific evidence on the use of music as an intervention for reducing psychological distress in cancer patients. The findings are expected to contribute to the strengthening of clinical practice, particularly in oncology nursing, by promoting more humanized and evidence-based care. Furthermore, the results may guide the development of care protocols and help identify knowledge gaps that can inform future research on the topic.

## Supporting information

S1 Fig Study selection flow diagram (adapted from the Preferred Reporting Items for Systematic Reviews and Meta-Analyses extension for Scoping Reviews, PRISMA-ScR). (TIF)

S2 Table Example of search strategy in the PubMed database. (DOCX)

S3 Checklist PRISMA-P (Preferred Reporting Items for Systematic reviews and Meta-Analyses Protocols) 2015 Checklist. (DOCX)

## References

[1] Guerra-Martín MD, Tejedor-Bueno MS, Correa-Casado M. Effectiveness of Complementary Therapies in Cancer Patients: A Systematic Review. Int J Environ Res Public Health. 2021;18(3):1017. doi: 10.3390/ijerph18031017 33498883 PMC7908482

[2] Bradt J, Dileo C, Myers-Coffman K, Biondo J. Music interventions for improving psychological and physical outcomes in people with cancer. Cochrane Database Syst Rev. 2021;10(10):CD006911. doi: 10.1002/14651858.CD006911.pub4 34637527 PMC8510511

[3] Silveira FM, Wysocki AD, Mendez RDR, Pena SB, Santos EM dos, Malaguti-Toffano S, et al. Impacto do tratamento quimioterápico na qualidade de vida de pacientes oncológicos. Acta Paulista de Enfermagem. 2021;34. doi: 10.37689/acta-ape/2021ao00583

[4] Costa GJ, Mello MJG de, Bergmann A, Ferreira CG, Thuler LCS. Tumor-node-metastasis staging and treatment patterns of 73,167 patients with lung cancer in Brazil. J Bras Pneumol. 2020;46(1):e20180251. doi: 10.1590/1806-3713/e20180251 31967271 PMC7462681

[5] Nobre SV, Arregi MMU, Moreira TMM, Pinto AGA, Silva MGC da. Therapeutic modalities in the first cancer treatment: percentage variation before and during the pandemic, in Ceará, a state in the Northeast region of Brazil. Rev Bras Epidemiol. 2025;28:e250024. doi: 10.1590/1980-549720250024 40366936 PMC12068809

[6] Stanczyk MM. Music therapy in supportive cancer care. Rep Pract Oncol Radiother. 2011;16(5):170–2. doi: 10.1016/j.rpor.2011.04.005 24376975 PMC3863265

[7] Gautama MSN, Haryani H, Huang T-W. Efficacy of smartphone-based virtual reality relaxation in providing comfort to patients with cancer undergoing chemotherapy in oncology outpatient setting in Indonesia: protocol for a randomised controlled trial. BMJ Open. 2023;13(7):e074506. doi: 10.1136/bmjopen-2023-074506 37491084 PMC10373714

[8] Rha SY, Lee J. Stable Symptom Clusters and Evolving Symptom Networks in Relation to Chemotherapy Cycles. J Pain Symptom Manage. 2021;61(3):544–54. doi: 10.1016/j.jpainsymman.2020.08.008 32828931

[9] Yang T, Wang S, Wang R, Wei Y, Kang Y, Liu Y, et al. Effectiveness of five-element music therapy in cancer patients: A systematic review and meta-analysis. Complement Ther Clin Pract. 2021;44:101416. doi: 10.1016/j.ctcp.2021.101416 34020291

[10] Nguyen KT, Hoang HTX, Bui QV, Chan DNS, Choi KC, Chan CWH. Effects of music intervention combined with progressive muscle relaxation on anxiety, depression, stress and quality of life among women with cancer receiving chemotherapy: A pilot randomized controlled trial. PLoS One. 2023;18(11):e0293060. doi: 10.1371/journal.pone.0293060 37922279 PMC10624313

[11] Yang H-F, Chang W-W, Chou Y-H, Huang J-Y, Liao Y-S, Liao T-E, et al. Impact of background music listening on anxiety in cancer patients undergoing initial radiation therapy: a randomized clinical trial. Radiat Oncol. 2024;19(1):73. doi: 10.1186/s13014-024-02460-3 38862982 PMC11167881

[12] Gezgin Yazıcı H, Ökten Ç, Karabulut E, Aliustaoğlu M. Effect of Music on Anxiety and Fatigue in Cancer Patients Undergoing Chemotherapy: A Randomized Controlled Trial. Arch Iran Med. 2024;27(11):611–7. doi: 10.34172/aim.31258 39534995 PMC11558607

[13] Santos KVGD, Dantas JKDS, Fernandes TE de L, Medeiros KS de, Sarmento ACA, Ribeiro KRB, et al. Music to relieve pain and anxiety in cardiac catheterization: A systematic review and meta-analysis. Heliyon. 2024;10(13):e33815. doi: 10.1016/j.heliyon.2024.e33815 39044980 PMC11263635

[14] Bradt J, Dileo C, Myers-Coffman K, Biondo J. Music interventions for improving psychological and physical outcomes in people with cancer. Cochrane Database Syst Rev. 2021;10(10):CD006911. doi: 10.1002/14651858.CD006911.pub4 34637527 PMC8510511

[15] Santos KVG dos, Rocha MA, Dantas JK dos S, Araújo SCM de, Dantas DV, Dantas RAN. Estratégias não farmacológicas na analgesia de adultos e idosos em procedimentos endovasculares: revisão de escopo. Rev Bras Enferm. 2022;75(suppl 4). doi: 10.1590/0034-7167-2021-0741pt 35946724

[16] Santos MSD, Thomaz F de M, Jomar RT, Abreu AMM, Taets GGDCC. Music in the relief of stress and distress in cancer patients. Rev Bras Enferm. 2021;74(2):e20190838. doi: 10.1590/0034-7167-2019-0838 34037140

[17] Silva LC de MA, Dos Santos KVG, Dos Santos JJ de S, Camara RP de POA, Bezerra E Silva SY, Silva HMMD, et al. Efficacy of aromatherapy with Lavandula angustifolia oil on postoperative pain after cardiac surgery: A randomized clinical trial. Explore (NY). 2024;20(6):103034. doi: 10.1016/j.explore.2024.103034 39032323

[18] Tricco AC, Lillie E, Zarin W, O’Brien KK, Colquhoun H, Levac D, et al. PRISMA Extension for Scoping Reviews (PRISMA-ScR): Checklist and Explanation. Ann Intern Med. 2018;169(7):467–73. doi: 10.7326/M18-0850 30178033

[19] Peters MDJ, Marnie C, Tricco AC, Pollock D, Munn Z, Alexander L, et al. Updated methodological guidance for the conduct of scoping reviews. JBI Evid Synth. 2020;18(10):2119–26. doi: 10.11124/JBIES-20-00167 33038124

[20] Arksey H, O’Malley L. Scoping studies: towards a methodological framework. Int J Soc Res Methodol. 2005;8(1).

[21] Bradbury-Jones C, Aveyard H, Herber OR, Isham L, Taylor J, O’Malley L. Scoping reviews: the PAGER framework for improving the quality of reporting. Int J Soc Res. 2022;25(4):457–70.

[22] Zhong C, Luo X, Tan M, Chi J, Guo B, Tang J, et al. Digital Health Interventions to Improve Mental Health in Patients With Cancer: Umbrella Review. J Med Internet Res. 2025;27:e69621. doi: 10.2196/69621 39984165 PMC11890151

[23] Mendes F de C de O, Santos KVGD, Silva TTM da, Pereira VDSL, Diniz KD, Ribeiro KRB, et al. Using music to reduce anxiety and stress in patients undergoing coronary angioplasty: A randomized clinical trial protocol. MethodsX. 2024;13:103064. doi: 10.1016/j.mex.2024.103064 39698490 PMC11652734

[24] Mendes F de C de O, Santos KVGD, Dantas JKDS, Araújo SCM de, Teixeira F de C, Leal KC da S, et al. Non-pharmacological strategies to reduce stress and anxiety in endovascular procedures: A scoping review. Nurs Open. 2024;11(3):e2105. doi: 10.1002/nop2.2105 38520118 PMC10960161

[25] Huda N, Banda KJ, Liu A-I, Huang T-W. Effects of Music Therapy on Spiritual Well-Being among Patients with Advanced Cancer in Palliative Care: A Meta-Analysis of Randomized Controlled Trials. Semin Oncol Nurs. 2023;39(6):151481. doi: 10.1016/j.soncn.2023.151481 37541810

[26] Xu Z, Liu C, Fan W, Li S, Li Y. Effect of music therapy on anxiety and depression in breast cancer patients: systematic review and meta-analysis. Sci Rep. 2024;14(1):16532. doi: 10.1038/s41598-024-66836-x 39019965 PMC11255342

