# Supplementary material for: The use of music in nursing care for psychological distress reduction in cancer patients: A scoping review protocol
Source: PLoS One. 2025 Nov 24;20(11):e0335895. doi: 10.1371/journal.pone.0335895 (PMC12643286; doi:10.1371/journal.pone.0335895)
Supplement: S2 Table — (DOCX) [file pone.0335895.s002.docx]

**Table 1**. Search strategy in PubMed PMC. Natal, RN, Brasil, 2025.

| Database | Search Strategy |
| --- | --- |
| Pubmed PMC | ((((((Music therapy) AND (Psychological Distress))) OR (Anxiety)) OR (Subjective Stress)) AND (cancer)) AND (nursing care) |

Source: Authors, 2025.
